# Melting and Recrystallization of Copper Nanoparticles Prepared by Microwave-Assisted Reduction in the Presence of Triethylenetetramine

**DOI:** 10.3390/ma13071507

**Published:** 2020-03-26

**Authors:** Li-Cheng Jheng, Yen-Zen Wang, Wen-Yao Huang, Ko-Shan Ho, Cheng-Hsien Tsai, Ching-Tang Huang, Huang-Shian Tsai

**Affiliations:** 1 Department of Chemical and Materials Engineering, National Kaohsiung University of Science & Technology, 415 Chien-Kuo Rd., Kaohsiung 80782, Taiwan; lcjheng@nkust.edu.tw (L.-C.J.); loe@nkust.edu.tw (C.-H.T.); 2 Department of Chemical and Materials Engineering, National Yun-Lin University of Science and Technology, Yun-Lin 64002, Taiwan; 3 Department of Photonics, National Sun Yat-sen University, 70 Lienhai Rd., Kaohsiung 80424, Taiwan; wyhuang@faculty.nsysu.edu.tw; 4 Taiwan Textile Research Institute, 20, Kejia Rd., Douliou City, Yun-Lin 64057, Taiwan; dhr@yuntech.edu.tw (C.-T.H.); tst@yuntech.edu.tw (H.-S.T.)

**Keywords:** microwave-assisted, nanocopper, triethylene tetramine, melting, recrystallization, chelation

## Abstract

The small sized copper nanoparticles (Cu-NPs), prepared in the presence of triethylene tetramine (TETA) and assisted with microwave irradiation, have an extremely low melting temperature. Melting of the small sizezd Cu-NPs can be triggered by the heat generated from the e-beam irradiation during SEM and TEM image construction. The dispersed Cu atoms around the agglomerated big Cu particles can undergo recrystallization immediately due to the strong driving force of the huge temperature difference to normal melting temperature (T_m_ = 1085 °C). Some of the Cu-NPs with bigger sizes also recrystallize and agglomerate into dense, big particles. According to X-ray diffraction patterns, these particles can agglomerate into compact, ordered Cu crystals in less than five minutes at 60 °C. The melting and recrystallization related endothermic and exothermic phase transitions of Cu-NPs can be found from differential scanning calorimeter (DSC) thermograms and optical microscopic pictures.

## 1. Introduction

Super tiny metal nanoparticles (NPs) with sizes less than 10 nm have proved useful in lots of areas in materials science and engineering due to their specific properties seldom found in other NPs with bigger sizes. The melting points of some metal NPs can be as low as 53.9 °C (<100 °C) [1,2], which was demonstrated directly from a differential scanning calorimeter (DSC) thermogram. However, the melting points of regular metals are usually higher than 500 °C. After depressing the melting point to below 100 °C, the metal can be processed in an easier way. Cu-NPs with sizes below 100 nm can be prepared with MW (microwave) irradiation [3,4] with or without the polyol [5,6,7] and reducing agents [8,9,10,11,12,13,14,15]. Especially for cheap, highly conductive Cu-NPs with smaller sizes [16,17,18,19,20,21,22,23,24,25,26,27,28,29,30], they are dispersible in solvents due to their super high surface area/weight ratio and can be mixed with resin into conducting Cu-ink. Therefore, it is possible to provide a continuous, highly conductive route for printed circuit formation after structural melting and particle recrystallization (agglomeration) of the Cu-NP ink, which can print directly on substrates and create continuous conducting circuit-patterns at temperatures slightly higher than RT (room temperature).

The thermal behaviors of the NPs are different from the bulk metals due to the big surface-to-volume ratio. The melting points (T_m_) of metal NPs are strongly size–dependent since the radius of the NPs is inversely proportional to the surface to volume ratio. When the number of the surface atoms surpasses or is comparable to that of the bulk, the T_m_ can decrease significantly. The surface-atoms are usually randomly distributed and loosely bonded. They are prone to run away from the NPs when little energy perturbation is provided from the thermal heating. In other words, surface melting (coagulation) can occur at temperatures lower than the regular T_m_. Theoretically, NPs with a size of 5 nm can cause a significant depression of T_m_ to several hundred degrees for some metals [31,32,33]. When the size is below 3 nm, the T_m_ drops drastically to below 100 °C [31,32,33,34] due to the presence of numbers of randomly distributed (non-crystallized) surface atoms. Zola [35] etc. was dealing with the preparation of Co-NPs with several methods. Less attention was paid to the T_m_ depression of Cu-NPs, and scarce literatures demonstrated the melting point below 100 °C directly by their thermograms or X-ray diffraction patterns for small sized Cu-NPs. The freer surface-atoms of small sized Cu-NPs can be released at temperatures far below normal T_m_ and solidify immediately due to the strong driving force derived from the huge temperature difference to normal T_m_, which can induce coagulation and agglomeration, resulting in the formation of new bigger particles.

Compared to the hydrothermal heating method, which consumes much energy during the redox reaction, the amino groups of triethylene tetramine (TETA) are able to capture Cu^2+^ ions by chelation and absorb MW to start the redox reaction immediately after MW exposure. The swiftly formed Cu particles from the irradiation of MW do not develop easily into large crystals since only some Cu^2+^ ions are enclosed by the chelating TETAs before MW irradiation. Besides, the exposure time of MW irradiation was very short, and the heating effect and melting/recrystallization were stopped immediately after the turn-off of the MW oven. It provides us a possible way to prepare Cu-NPs with sizes below 3 nm in a facile way by direct MW irradiation in the presence of TETAs.

In the following studies, we will apply the MW irradiation to trigger the redox reaction between Cu^2+^ and TETA to prepare Cu-NPs with small sizes. The melting and recrystallization phenomena of Cu-NPs will be investigated by SEM and TEM. The melting and recrystallization temperatures will be directly measured by DSC, X-ray diffraction, optical microscopy, and the mechanism will be studied by electron microscopy.

## 2. Materials and Methods

### 2.1. Preparation of Cu-NPs

**Figure 1:**
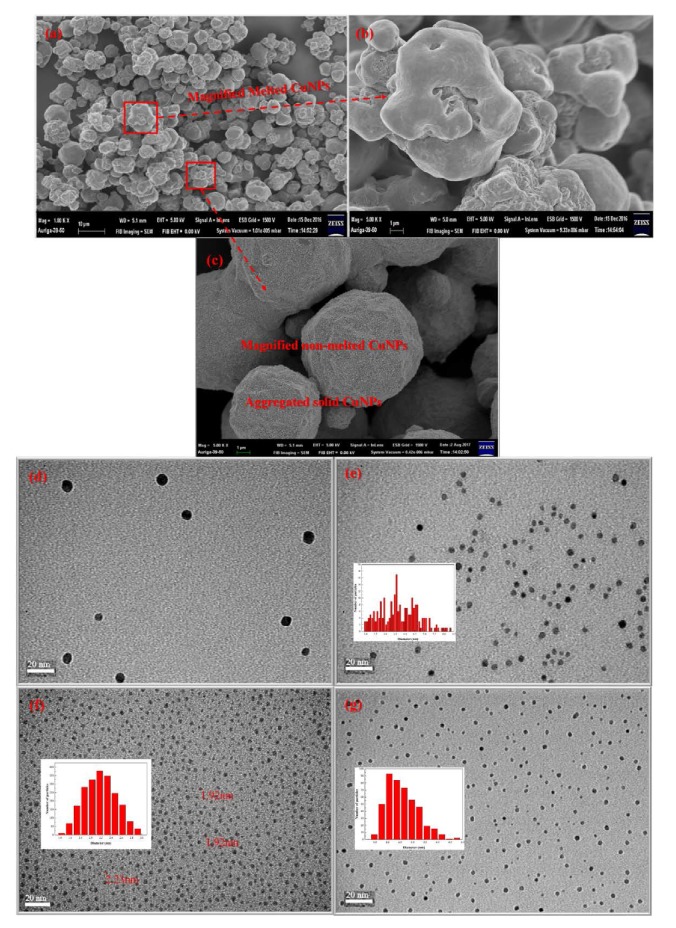
Different magnifications of SEM micrographs of Cu-NPs prepared from MW irradiation in the presence of TETA (**a**–**c**); TEM micrographs of TETA/Cu^2+^ prepared with different ratios (**d**) 1.5/1 (**e**) 2.0/1 (**f**) 2.5/1 (**g**) 3.0/1.

The typical preparation of small sized Cu-NPs is: 0.010 mol (2.49 g) CuSO_4_∙5H_2_O (NIPPON SIYO, Tokyo, Japan) was dispersed in 5.59 mL (6.2 g, 0.1 mol) EG (J.T. Baker, PA, USA). The color of the blue Cu^2+^/EG solution became darker after chelating with 0.025 mol (3.65 g) TETA (ACROS, Brussel, Belgium), demonstrated in Figure 1. The mixture was irradiated with the microwave oven (microwave oven, N-ST342, PANASONIC, Tokyo, Japan) for 100 s in an atmosphere of high purity N_2_. The power was set at 700 W, and temperature was controlled at below 100 °C. The color of the mixture turned from a dark blue to light brown color in less than a minute. The Cu-NPs were also prepared with different mole ratios of TETA/Cu^2+^ (1.5, 2.0, and 3.0), except 2.5 in the same conditions. A comparison experiment by direct heating Cu^2+^ in the EG medium (hydrothermal method) was carried out in the presence of TETA (TETA/Cu^2+^ = 1.5/1) for 2.5 h.

**Scheme 1:**
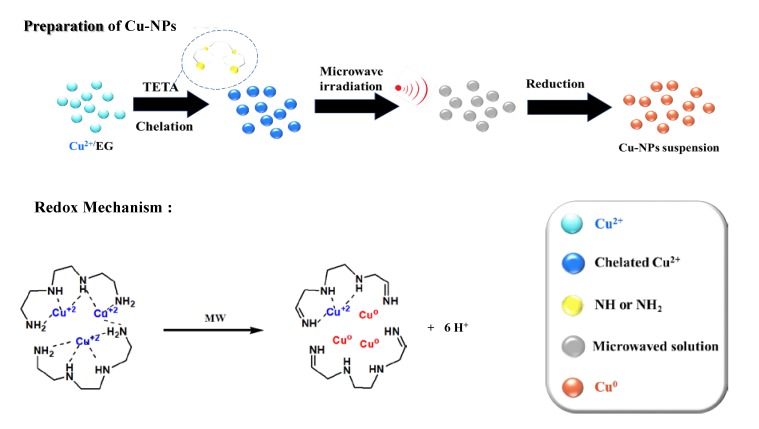
Schematic diagram and reaction mechanism of preparation of Cu-NPs before and after microwave (MW) irradiation.

The Cu-NPs were separated from the solvent medium and TETAs by centrifugation at 3000 rpm for 60 min in 15 min intervals for four times to avoid heat generation, followed by washing alternatively with ethanol (TEDIA, St. Louis, MO, USA) and de-ionized water several times. The washed Cu-NPs were then sonicated in de-ionized water for 1 min, before going into another centrifugation and washing cycle, which allowed us to collect Cu-NPs at the bottom and totally remove solvent (EG) and TETA. Eventually, the obtained Cu-NPs were dried in a vacuum oven at RT for one day before being stored in an ice-box. [31,36] The schematic diagram of the preparation is demonstrated in Scheme 1.

### 2.2. Characterization

All samples were kept in an ice-box before measuring and during transportation. The size, size distribution, and morphologies of Cu-NPs were characterized by SEM (Field emission gun scanning electron microscope, AURIGAFE, Zeiss, Oberkochen, Germany) and TEM (Cryo-transmission electron microscope, JEM-1400, JEOL, Tokyo, Japan). The X-ray diffraction patterns were obtained from a copper target (Rigaku) X-ray source with a wavelength of 1.5402 Å. The melting and recrystallization temperatures of Cu-NPs were measured directly from the DSC thermogram with MDSC2920, TA INSTRUMENT. The thermal stability of Cu-NPs was evaluated by Thermogravimetric Analysis (TGA) (TA SDT-2960, Austin, TX, USA) thermograms. The weight variation was recorded from RT to 800 °C at 10 °C min^−1^ in purging air. The melting/recrystallization process was also illustrated with optical microscopic pictures taken in a hot stage of Zeiss Axioscope 50.

## 3. Results and Discussion

### 3.1. Microwave Irradiation

Light blue Cu^2+^ EG (ethylene glycol)-solution became dark blue immediately after the introduction of TETA. The dark blue color originated from the chelation with TETA, as illustrated in the lower part of Scheme 1. Exposed to MW irradiation, the dark blue solution became red and tiny Cu particles were found to uniformly suspend in the solution. Red Cu elements were not easily obtained unless the Cu^2+^/EG solution was refluxed at 120 °C for several hours in the absence of TETA. A comparison experiment assisted with MW irradiation for Cu^2+^/EG solution in the absence of TETA demonstrated no red Cu particles and remained dark blue in color. Only flake-like materials were present, which were characterized by X-ray diffraction (XRD) pattern as crystalline of Cu_4_SO_4_(OH)_6_ [31]. It indicated EG was not a good reducing agent, and the reducing can only be started at a high temperature like 120 °C. However, EG is a good solvent for CuSO_4_ solids, and can allow Cu^2+^ to complex with TETAs in this system. On the other hand, the dielectric constant of TETA is 11.4, which can effectively absorb MW and transfer it into frictional heat by performing molecular rotation. The generated frictional heat can ignite the redox reaction, resulting in the formation (reduction) of red Cu-NPs.

### 3.2. SEM and TEM

The morphologies of the Cu-NPs obtained from MW irradiation can be observed from the SEM micrographs demonstrated in Figure 1. A lot of copper microparticles (Cu-MPs) with diameters around 5–10 μm are seen in Figure 1a. Surprisingly, some of the particle surfaces became blurred (melted) during SEM scanning. Similar melting phenomenon and mechanism were also found for various metal particles [36,37,38,39,40,41,42]. After magnifying the blurred area to 10 times (Figure 1b), the surface melting phenomenon was clearly observed. SEM scanning is performed by projecting an e-beam on the samples, which can also generate heat on the sample during image construction. However, the generated thermal energies cannot be over 1000 °C to induce the melting of Cu crystals (T_m_ ≈ 1085 °C). The only reasonable explanation for such low temperature melting, is that the surfaces of the obtained Cu-MPs are actually composed of lots of small sized Cu-NPs whose loose structures allow them to melt at temperatures well below the normal melting point. In fact, what we obtained after swift MW irradiation in the presence of TETA is Cu-NPs with size below 10 nm and are likely to melt from the heat generated during image construction. To confirm the presence of small sized Cu-NPs, the image of the non-melting area in Figure 1a was magnified 100 times and illustrated in Figure 1c. The surfaces of the particles found in Figure 1a are actually made of the aggregated Cu-NPs with sizes below 10 nm, which can melt upon the irradiation of the e-beam.

The size of the Cu-NP was found to be strongly dependent on the concentration of TETA, which behaves as a reducing center during MW irradiation. Various Cu-NPs were prepared with different TETA/Cu^2+^ ratios by MW irradiation, and the sizes and size distributions of the obtained Cu-NPs were examined by TEM micrographs, as illustrated in Figure 1d–g.

In Figure 1d, the size of Cu-NPs prepared with TETA/Cu^2+^ = 1.5/1 is around 5 nm. Theoretically, the average size of the obtained Cu-NPs could narrow down when more reducing agents were present when the concentration of Cu^2+^ remains the same. Figure 1e,f show the average size of Cu-NPs below 3 nm when the molar ratios of TETA/Cu^2+^ were higher than 1.5/1. The sizes of the obtained Cu-NPs also depend on the coordination (chelation) patterns between Cu^2+^ and TETA, as one of the patterns is depicted in Scheme 1. Since lower numbers of Cu^2+^ can be chelated with every TETA when its concentration is increased, the average size of Cu-NPs obtained with 2.0/1 ratio rationally decreased from 5 nm to 3.7 nm according to Figure 1e, where the most probable size is 3.5 nm based on the inset diagram, Figure 1e. When the ratio of TETA/Cu^2+^ was adjusted to 2.5/1, the size of almost every Cu-NP was smaller than 3 nm, and the average size decreased to 2.2 nm, as observed in Figure 1f. When ratio increases to 3/1, the average size of Cu-NPs increased to 2.70 nm, as shown in Figure 1g. Even though the most probable size at this ratio is still below 3 nm, lots of particles with sizes well above 3 nm are formed, indicating excessive TETAs can, on the contrary, increase the average size of Cu-NP particles. Consequently, the optimal molar ratio for preparation of Cu-NPs with the smallest size (below 3 nm) is 2.5/1 when Cu-NPs were obtained in EG solvent assisted with MW irradiation. The following discussions will concentrate on the Cu-NPs prepared with this ratio (2.5/1).

**Figure 2:**
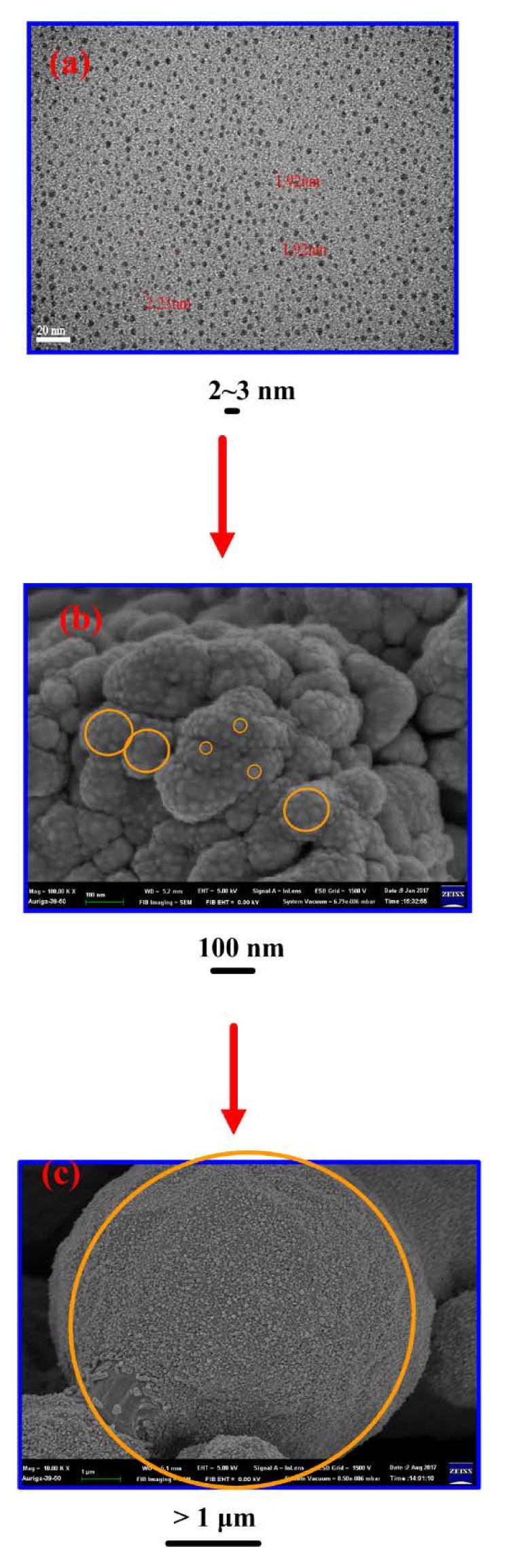
(**a**) TEM micrograph of Cu-NPs with small sizes, (**b**) SEM micrograph of aggregated Cu-NPs (Cu-MPs), and (**c**) SEM micrograph of large-scale aggregated Cu-NPs (Cu-MPs).

Some of the micrographs were chosen to illustrate the aggregated phenomenon of Cu-NPs in Figure 2. The aggregation of Cu-NPs results in the formation of the bigger particles of about 100 nm (Figure 2b). Even larger particles with sizes larger than 1 μm (Cu-MPs) can be observed in Figure 2c. We realize that the aggregated phenomenon found from Figure 2a–c is natural for the NPs to reduce the surface area by agglomeration. In other words, the bigger particles observed in Figure 2b,c are actually made of self-assembled Cn-NPs.

**Figure 3:**
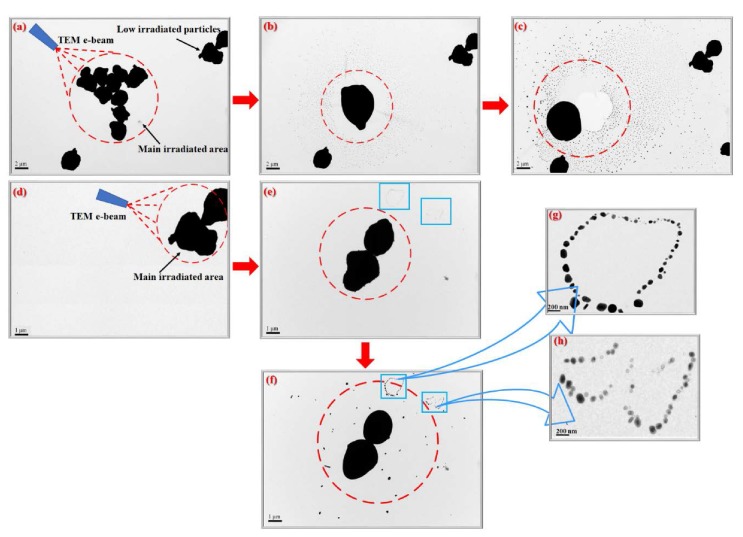
TEM micrographs of Cu-MPs prepared with molar ratio of TETA/Cu^2+^ = 2.5/1 with different exposure times and irradiated areas: (**a**) in the beginning, (**b**) at 5 s, and (**c**) at 10 s; (**d**) magnified right-top area of (**a**), (**e**) 5 s, (**f**) 10 s later; (**g**,**h**) insets of two chosen magnified areas of (**f**).

The heavily irradiated area by longer e-beam exposure is marked with dashed red circles in Figure 3. Melting occurred only within heavily exposed areas (similar to focused area in optical microscopy), as observed from Figure 3b,c. The particles in the less irradiated area (outside the focused area) remain intact, without significant melting or fusion. During the initial stage of TEM scanning, neither melting nor tiny dots can be perceived in Figure 3a. However, several aggregated particles within the highly irradiated area started to melt and impinge (agglomerate) into one big particle with smooth surface in a very short time, as demonstrated in Figure 3b.

More and more tiny nanodots also appear around the melting particles in the later stage due to gradual recrystallization, as shown in Figure 3b,c. It is possible that some of the surface atoms of particles were released during melting and diffused to the surrounding area, followed by recrystallization into new Cu-NPs around the melting big particle. It was also found that closer to the melting particle, smaller tiny particles were present (Figure 3c).

Similar melting and recrystallization occured when we focused the e-beam on the upper right two-particle and increased the magnifications. Interestingly, the touched two-particle located in the upper right area did not go on melting during the image construction period of Figure 3a–c. It started to melt when focused. The two-particle had a very rough surface (Figure 3d) at the beginning of focusing and started to melt after focusing (Figure 3e). The two-particle never impinged into one particle but became a hard solid eventually after long term exposure. The surface smoothening effect was also observed during image construction, and many tiny particles (≤100 nm) also appeared in the neighboring area and was demonstrated in Figure 3e,f.

Chain-like archipelagos of Cu-NPs (Figure 3e,f) gradually emerge in the neighborhood close to the focused (irradiated) area. These new coral reef-like Cu-NPs demonstrate no significant melting during e-beam irradiation, indicating they are normal crystallized Cu-NPs (Figure 3g,h) obtained from recrystallization. The shining surfaces of the Cu-NPs in Figure 3g,h resemble diamond stones, indicating that they are highly crystallized particles.

**Figure 4:**
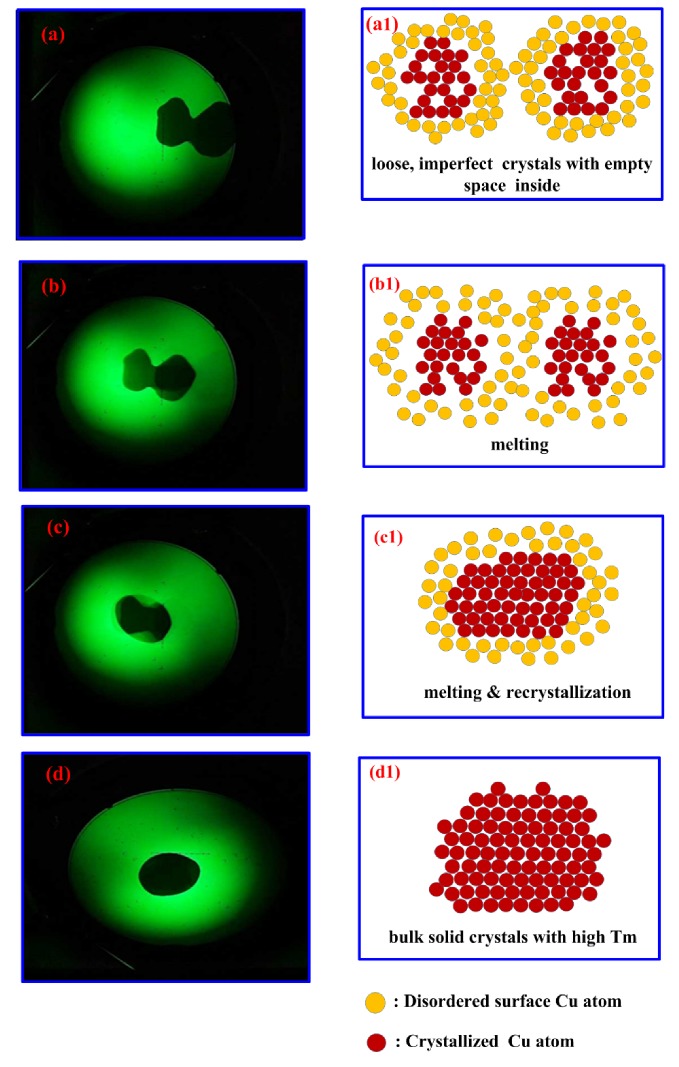
(**a**–**d**) Pictures taken directly from the monitor screen of TEM; (**a1**–**d1**) Schematic diagrams depicting the melting and recrystallization (agglomeration and impingement) of two individual particles upon exposure to TEM e-beam.

Schematic diagrams that depict the e-beam induced melting/recrystallization of Cu-NPs or Cu-MPs were drawn and put side by side with the pictures taken directly from the TEM monitor screen, as illustrated in Figure 4. Figure 4a reveals two impinging Cu particles carrying out fusion upon e-beam irradiation during image construction, as depicted in Figure 4a1. These two particles with micro-sizes are actually made of imperfect crystals with disordered Cu atoms on the surface (more) and inside (less). When the exposure time is increased (Figure 4b,b1), the particles impinge further and the centers of these two particles come closer. The impingement and following fusion was enhanced when exposure was further increased (Figure 4c). Significant bulk (inside) atom rearrangements (recrystallization) were believed to appear like in Figure 4c1, which demonstrates compact and perfect crystallinity, gradually created by consuming bulk disordered atoms. Eventually, after longer exposure, the surface atoms join the bulk crystallization and only one single particle is observed on the screen (Figure 4d). A robust solid-state crystal, described in Figure 4d1, is formed and no further melting can be observed, since its melting point is returned to normal at around 1085 °C after recrystallization.

### 3.3. Wide Angled X-ray Diffraction (WXRD)

**Figure 5:**
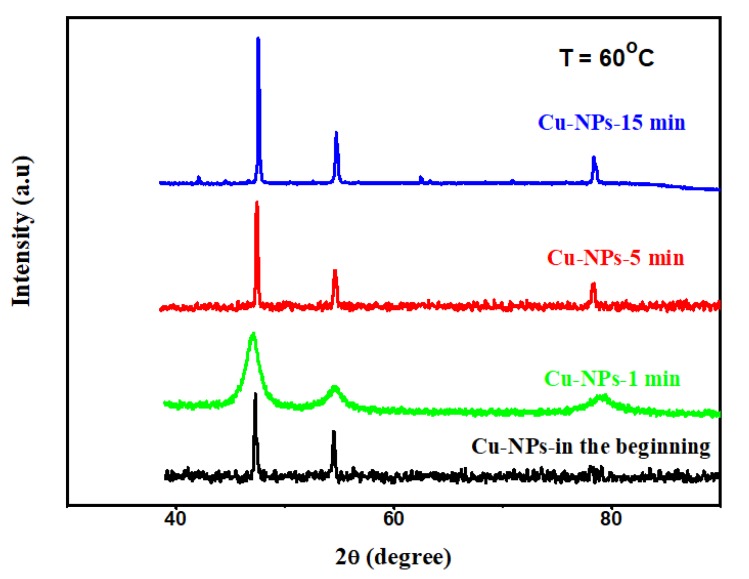
X-ray diffraction patterns of small sized Cu-NPs after staying at 60 °C for different times.

The neat small sized Cu-NPs prepared from the MW irradiation are structurally instable and can easily melt and recrystallize into compact crystals by slight thermal perturbation during the image construction of SEM and TEM. The X-ray diffraction (XRD) patterns of the small sized Cu-NPs, which stay for different times at 60 °C, can tell how long the Cu-NPs will go on structural reorganization and reordering. Fresh samples demonstrated the same pattern as common Cu crystals, with significant peaks of 100, 200, and 220, according to Figure 5. Surprisingly, the sample started to melt (coagulate) in less than 1 min at 60 °C, and a loose crystalline pattern was observed in Figure 5. However, the loose crystalline structure, which was created by staying for 1 min at 60 °C, could originate from either melting or recrystallizing Cu-NPs. When the staying time was over 5 min at 60 °C, perfect X-ray diffraction patterns, like the common Cu crystalline structure, were observed. The perfect X-ray diffraction patterns were contributed by the agglomerated particles or the regenerated tiny crystallites found around the exposed big particles, under the irradiation of the e-beams of electron microscopies. The recrystallized Cu crystals have perfect crystalline structure with T_m_ equal to 1085 °C, and no further melting phenomena (broadened peaks) was found after a longer time at 60 °C.

### 3.4. Differential Scanning Calorimeter (DSC)

**Figure 6:**
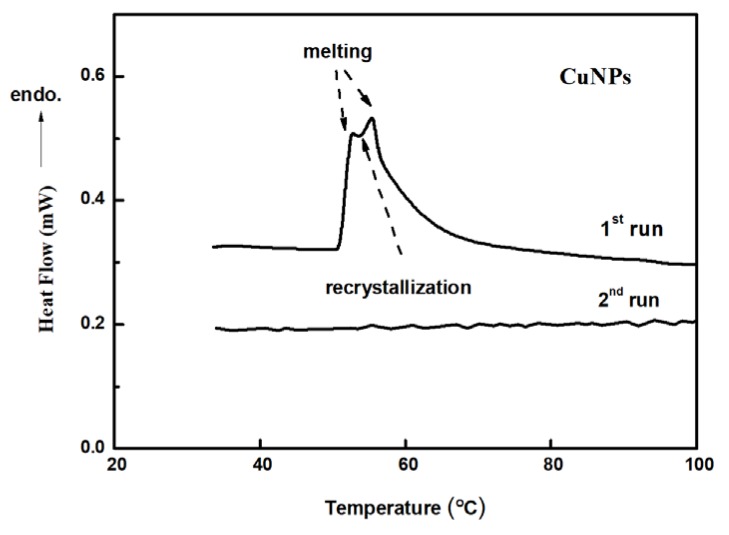
Differential scanning calorimeter (DSC) thermograms of Cu-NPs prepared with TETA/Cu^2+^ = 2.5/1.

The melting and recrystallization phenomena observed during SEM and TEM measurements reveal the low T_m_ of the small sized Cu-NPs, which was estimated to be around 60 °C by DSC thermogram. The phase transitional peaks could be directly observed from the thermogram obtained from the differential scanning calorimeter (DSC), running from 0 to 100 °C as illustrated in Figure 6. The DSC thermograms of Cu-NPs prepared with TETA/Cu^2+^ ratio equal to 2.5/1 (size below 3 nm) is demonstrated in Figure 6. The thermogram exhibits clear endothermic and exothermic peaks in the first heating run. No peak can be observed in the second heating run, revealing that recrystallization was already complete and the new formed crystals own T_m_ as high as 1085 °C, much higher than the peak temperatures found in the first run which cannot be measured. The reasonable explanation for the low T_m_ is that the clusters of small sized Cu-NPs [6] could be loose icosahedral or decahedral structures [12,19,20,43,44,45] which are able to dissociate or collapse at much lower temperatures than the normal T_m_.

Peaks disappeared in the second heating run, indicating that all the Cu-NPs have melted and have recrystallized into larger solid-state particles before 60 °C, before the second run. Since the T_m_ of the small sized Cu-NP is very close to 60 °C, it explains why the Cu-NPs can melt and agglomerate together into perfect crystals when exposed to e-beam during image construction (SEM and TEM).

### 3.5. Thermogravimetric Analysis (TGA)

To check the presence of residual TETA and the thermal stability of the Cu-NPs prepared with the assistance of MW in the presence of TETA, we carried out the thermal heating in the purging air.

**Figure 7:**
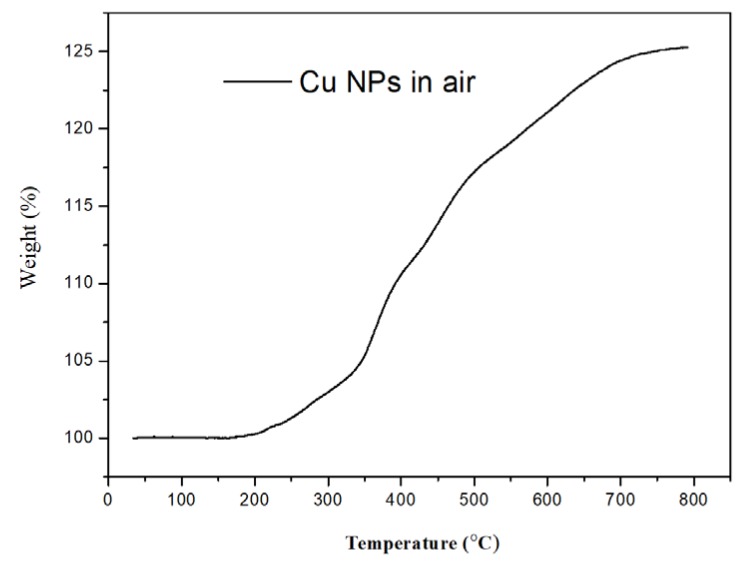
Thermogravimetric Analysis (TGA) thermogram of Cu-NPs in purging air (prepared with TETA/Cu^+2^ ratio equals to 2.5/1).

No significant weight change was found until 180 °C in the air for TGA thermogram demonstrated in Figure 7. It indicates that most of the residual TETAs were removed by washing with water and ethanol alternatively. The Cu-NPs illustrated significant oxidation and became Cu_2_O or CuO only after 200 °C. It confirmed that the prepared Cu-NPs good thermal stability in the air.

### 3.6. Optical Microscopy

**Figure 8:**
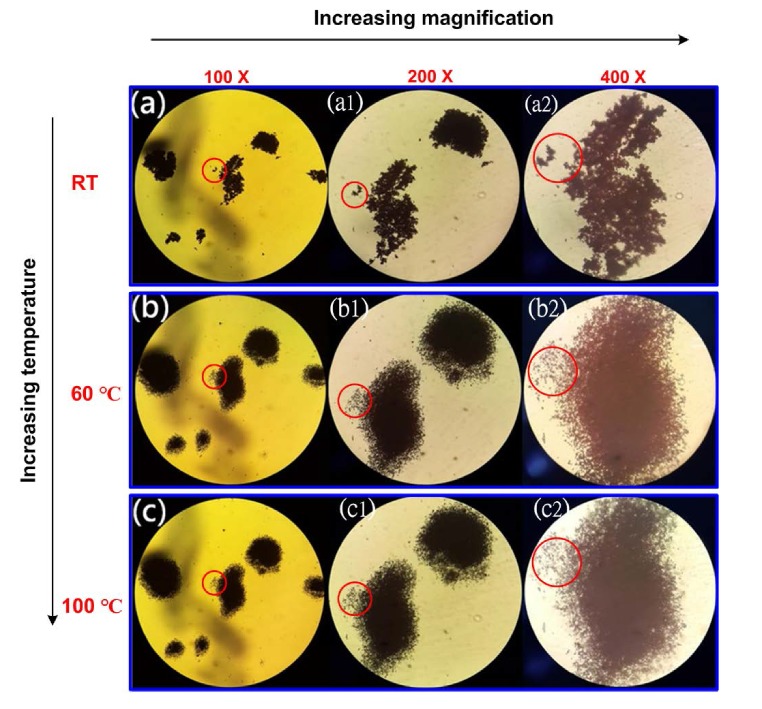
Optical microscopic pictures of Cu-NPs taken at (**a**) RT, (**b**) 60 °C, and (**c**) 100 °C, with 100×, 200×, and 400× magnifications, respectively.

The fusion and recrystallization were also found under optical microscopy by demonstrating pictures taken at different temperatures in a hot stage. The pictures were taken at different temperatures with various magnifications, and the areas with significant morphological variation due to temperature rising are circled in red. In Figure 8a,a1,a2 images, Cu particles are seen at 100×, 200×, and 400× magnification, respectively. These particles demonstrate no significant melting or recrystallization at RT. When temperature was increased to 60 °C, the large particles within the red circle marked areas disappeared, and new small particles formed. It seems the overlapped, disordered particles melted in the beginning and recrystallized into small, separated particles at 60 °C, as shown in Figure 8b,b1,b2. In fact, the melting and following recrystallization are found in every part of the sample, indicating most of the particles have very low T_m_ close to 60 °C. After staying at 60 °C for a while, the temperature of the hot stage was further increased to 100 °C. However, pictures taken at all magnifications do not demonstrate any significant morphological variation with pictures illustrated in Figure 8c,c1,c2. All Cu-NPs are believed to solidify (recrystallized solid) during temperature holding at 60 °C, and neither melting nor recrystallization occurs at 100 °C.

## 4. Conclusions

The small sized Cu-NPs prepared by microwave irradiation in the presence of TETA is confirmed directly by the SEM and TEM micrographs. The melting and following recrystallization processes of the Cu-NPs are clearly monitored by e-beam exposure from electron microscopies during measuring. All of them demonstrated that the particles after melting/recrystallization had firm, solid-state crystalline structures, and did not dissociate further in the second heating or at much higher temperatures since the T_m_ already reverted to 1085 °C. The inferiority of these Cu-NPs is that only separate isolated particles obtained after thermal heating, which cannot provide a continuous, highly conductive route for printed circuit formation after structural melting and particle recrystallization. In the future, we will concentrate on preparing Cu-NPs that can print directly on substrates, and create continuous conductive circuit-patterns at temperatures slightly higher than RT.
